# Enhancing Cell Proliferation and Osteogenic Differentiation of MC3T3-E1 Pre-osteoblasts by BMP-2 Delivery in Graphene Oxide-Incorporated PLGA/HA Biodegradable Microcarriers

**DOI:** 10.1038/s41598-017-12935-x

**Published:** 2017-10-02

**Authors:** Chuan Fu, Xiaoyu Yang, Shulian Tan, Liangsong Song

**Affiliations:** 1 Department of Hand and Foot surgery, The First Hospital of Jilin University, Xinmin Street No. 71, Changchun, TX, 130021 P.R. China; 2 The First Hospital and Institute of Immunology, the First Hospital of Jilin University, Xinmin Street No. 71, Changchun, TX, 130021 P.R. China; 3 Department of Orthopedic Surgery, the Second Hospital of Jilin University, Ziqiang Street No. 218, Changchun, TX, 130041 P.R. China

## Abstract

Lack of bioactivity has seriously restricted the development of biodegradable implants for bone tissue engineering. Therefore, surface modification of the composite is crucial to improve the osteointegration for bone regeneration. Bone morphogenetic protein-2 (BMP-2), a key factor in inducing osteogenesis and promoting bone regeneration, has been widely used in various clinical therapeutic trials. In this study, BMP-2 was successfully immobilized on graphene oxide-incorporated PLGA/HA (GO-PLGA/HA) biodegradable microcarriers. Our study demonstrated that the graphene oxide (GO) facilitated the simple and highly efficient immobilization of peptides on PLGA/HA microcarriers within 120 min. To further test *in vitro*, MC3T3-E1 cells were cultured on different microcarriers to observe various cellular activities. It was found that GO and HA significantly enhanced cell adhesion and proliferation. More importantly, the immobilization of BMP-2 onto the GO-PLGA/HA microcarriers resulted in significantly greater osteogenic differentiation of cells *in vitro*, as indicated by the alkaline phosphate activity test, quantitative real-time polymerase chain reaction analysis, immunofluorescence staining and mineralization on the deposited substrates. Findings from this study revealed that the method to use GO-PLGA/HA microcarriers for immobilizing BMP-2 has a great potential for the enhancement of the osseointegration of bone implants.

## Introduction

Large bone defects associated with trauma, tumuor, and infection frequently require surgical intervention. Bone grafts, including autografts, allografts, and xenografts, have been widely used to regenerate these defects^1^. However, traditional bone grafts have some drawbacks such as their limited supply, immune reaction and the transfer of pathogens, which have limited their development for bone defect repair^2–4^. Bone tissue engineering, which shows great potential in developing bone grafts to induce bone tissue regeneration, has thus attracted considerable attention. Cell microcarriers composed of biodegradable polymers, such as poly (lactide) (PLA), polycaprolactone (PCL) and poly (lactide-co-glycolide) (PLGA), are among the most effective candidates due to their unique properties for bone tissue regeneration^5–7^. Furthermore, the main advantage of biodegradable microcarriers is that they provide a large number of cells for the field of cell therapy and can be administered using 10 to 16-gauge needles at the bone defect site^8,9^. PLGA is an FDA-approved degradable materials with good mechanical properties, low immune-genicity and toxicity that frequently used to prepare cell microcarriers for bone repair^10–12^. However, the hydrophobicity of PLGA and the lack of cell recognition signals have restricted its application. To address these issues, a promising technique is bioactive minerals as fillers incorporated into PLGA polymer matrix is a promising technique. Hydroxyapatite (Ca_10_(PO_4_)_6_(OH)_2_, HA) is an effective component for biomimetic materials, which has good biocompatibility and osteoconductivity. Many studies have reported that the composites of PLGA and HA can markedly improve the osteogenic differentiation and mineralization of cells and provide more versatile applications than pure PLGA^13–15^. Therefore, the combination of HA and PLGA can be expected to attain the optimum bone grafts for bone tissue engineering.

In addition, the combination of growth factors into microcarriers can have the ability to improve the biological properties of microcarriers. Among growth factors, bone morphogenetic protein-2 (BMP-2) is a key factor in inducing osteogenesis and has been widely used in tissue engineering approaches for the repair of bone injuries and bone defects. Many studies have reported that polymer materials incorporated with BMP-2 can significantly induce cell osteogenic differentiation as well as enhance bone formation^16–18^. Furthermore, BMP-2 was approved by the US Food and Drug Administration (FDA) for clinical use to induce bone formation via the enhancement of osteoblast progenitor cell recruitment and angiogenesis^19^. The conventional method of the combination of BMP-2 into polymer materials is that growth factors are incorporated directly into the polymer materials during the polymer materials fabrication. However, because the fabrication of polymer materials must use organic solvents, the bioactivity of the growth factor will be damaged or reduced. Compared to these conventional methods, the immobilization method has attracted much attention as a new delivery method. More recently, a large number of methods for the uniformly immobilization of growth factors has been evaluated. These methods include plasma treatment, photoreactive gelatine, poly (dopamine) coating, and heparin-conjugated PLGA^20–23^. More importantly, the immobilized growth factors can be localized and retained in the designated location to maintain the stimulation effect for a long period. Graphene oxide (GO) nanosheets, derived from the oxidation of graphene, are single-layer, two-dimensional networks of sp2-and sp3-hybridized carbon atoms, which have attracted much attention because of their unique physicochemical characteristics, including extraordinary mechanical properties, excellent biocompatibility, hydrophilic functional groups, low toxicity and large loading capacity^24–26^. More importantly, GO is composed of hydrophobic π domains in the core region and ionized groups along the edges. These features enhance its binding affinity with BMP-2 via hydrophobic and electrostatic interactions^27,28^. The present study investigated the efficiency with which GO can carry and deliver BMP-2 for bone formation. La *et al*. successfully coated GO on the surface of titanium and the immobilized BMP-2 is retained in the designated location to maintain the stimulation effect for a long period^28^. Furthermore, La *et al*. also found that the BMP-2 immobilized GO/fibrin gel composites showed higher structural stability and bioactivity than BMP-2 immobilized fibrin gel^27^. Therefore, GO may be an effective carrier for BMP-2 delivery, which can not only reduce the usage of BMP-2, but also make the composites perform long-term osteoconductivity. Furthermore, previous studies demonstrated that the GO and HA showed the additive effect on enhancing osteogenesis, which can be effectively utilized as bone grafts for bone defect repair^29–31^.

Thus, in this study, we report graphene oxide-incorporated PLGA/HA microcarriers with BMP-2 immobilization to enhance the cell adhesion and osteogenic differentiation of MC3T3-E1 cells. The oxide-incorporated PLGA/HA microcarriers were fabricated by an emulsion–solvent evaporation method, and the BMP-2 was immobilized on the surface of the microcarriers by immersing the microcarriers in solutions of BMP-2. The improvement of hydrophilicity and MC3T3-E1 cells proliferation were investigated to evaluate the effect of GO on the PLGA/HA microcarriers. Then, alkaline phosphatase (ALP) activity, mineral deposition, and bone-related protein expression were performed to explore the osteoinductive effect of BMP-2 immobilized microcarriers. Our study may thus not only provide a potential biomaterial for bone tissues engineering but also contribute to a better understanding of the biological applications of engineered graphene-based nanomaterials.

## Materials and Methods

### Materials

PLGA (molecular weight = 80000, LA/GA = 75/25) was purchased from SinoBiomaterials Co., Ltd. BMP-2 was purchased from UB Biotech. HA powder was obtained from Nanjing Emperor Nano Material Co., Ltd. GO was purchased from Chengdu Organic Chemicals Co. Ltd, China (thickness: 0.55–1.2 nm diameter: 0.5–3 μm). Bovine serum albumin (BSA) was obtained from Beijing Solarbio Science & Technology Co., Ltd. Dichloromethane (DCM) was obtained from Beijing Chemical Works. 1,1,1,3,3,3-hexafluoroisopropanol (HFIP), 3-(4,5-dimethyl-2-thiazolyl)-2, 5-diphenyl-2- H-tetrazolium bromide (MTT) and BCA protein assay kit were purchased from Sigma-Aldrich (USA). Cetylpyridinium chloride (CPC) was purchased from Aladdin Chemistry Co. Ltd. The reagents for cell experiments were purchased from Gibco (USA).

### Preparation of Graphene Oxide-Incorporated PLGA/HA Microcarriers

Graphene oxide-incorporated PLGA/HA (GO-PLGA/HA) microcarriers were prepared via an emulsion–solvent evaporation method as previously described^32^. Briefly, 50 mg HA and 450 mg PLGA were dissolved in 9 mL DCM to prepare the PLGA/HA solution, and 10 mg GO were then dissolved in 1 mL HFIP to prepare the GO suspension. Then, the GO suspension was dropped into PLGA/HA solution and the resulting solution was emulsified with a homogenizer (Ultra-Turrax T-25 Basic, IKA) at 13,500 rpm for 5 min. The total solids content of HA and GO were 10% and 2% (w/w), respectively. The final mixture was poured into a rapidly stirring PVA solution (150 mL, 2% (w/v)) at 400 rpm and then stirred overnight to allow the solvent to evaporate. The PVA solution was decanted off and the microcarriers were washed three times in distilled water. Pure PLGA, and PLGA/HA microcarriers were also prepared under the same conditions.

### Surface immobilization of BMP-2

The graphene oxide-incorporated PLGA/HA microcarriers were placed in a 24-well plate. One millilitre of BMP-2 solution with different concentrations of 50, 100, and 500 ng·mL^−1^ in pH 7.4 phosphate buffer saline (PBS) was added into each well. The microcarriers were incubated in the BMP-2 solution for 2 h at room temperature on a shaker. The microcarriers were then washed with distilled water three times to remove the unattached peptides.

### Analyses of Microcarriers

The surface morphology and topography of the microcarriers was examined by a scanning electron microscope (SEM, XL 30 ESEM-FEG, FEI). The Fourier transform infrared spectroscopy (FT-IR, Perkin Elmer, FTIR-2000) and X-ray diffraction (XRD, D8 ADVANCE, Germany) were used to determine the chemical structure of the microcarriers.

Because of the size and geometry of microcarriers, the contact angle measurements on microcarries were made by relative measurements. PLGA, PLGA/HA and GO-PLGA/HA films were prepared and treated under similar conditions to the microcarriers. Static air–water contact angle measurements of the films were obtained using the sessile drop method on a contact angle system (VCA 2000, AST).

### Adsorption Rate of bovine serum albumin

Bovine serum albumin was selected as a model protein to determine the adsorption kinetics and adsorption efficiencies of synthesized microcarriers. The microcarriers were interacted with aqueous bovine serum albumin solutions by means of rotator. Briefly, 50 mg of the microcarriers were incubated with 10 mL of bovine serum albumin solution (2 mg·mL^−1^) under stirring at 150 rpm for 2 h. The amounts of bovine serum albumin loaded were determined through the concentration reduction of BSA within the samples using a BCA protein assay (wave length = 562 nm). Furthermore, the microcarriers were incubated on Rhodamine B labelled BSA (Sigma) solution (2 mg·mL^−1^) for 2 h. Then the samples were rinsed in PBS 3 times and mounted for visualization with a fluorescence microscope (TE2000-U, Nikon).

### Cell spreading, attachment, and proliferation assays

Cell experiments were performed by using mouse preosteoblast MC3T3-E1 cells purchased from Institute of Biochemistry and Cell Biology, Shanghai Institutes for Biological Sciences, Chinese Academy of Sciences. Cells were cultured with Dulbecco’s modified Eagle’s medium (DMEM, Gibco) supplemented with 10% FBS (Gibco), 10 mM HEPES (Sigma), 63 mg L^−1^ penicillin (Sigma) and 100 mg L^−1^ streptomycin (Sigma) in a humidified incubator at 37.8 °C and 5% CO_2_. Briefly, the microcarriers (PLGA, PLGA/HA, GO-PLGA/HA and BMP-2-immobilized microcarriers) were sterilized by immersing in 70% alcohol for 30 min. After being washed by PBS for three times and immersed in cell culture medium overnight, they were placed into 24-well plate (10 mg·well^−1^) to cover the bottom of wells. 1 mL MC3T3-E1 cell suspension (2.5 × 10^4^ cells·mL^−1^) was then seeded into each well. The plates were incubated at 37.8 °C in a humidified 5% CO_2_ atmosphere and the culture medium was replaced every 2d. For the cell proliferation examination, after 1, 4 and 7 d culture, 100 mL of MTT (5 g·L^−1^ in PBS) was added to each well and the cells were incubated for an additional 4 h. Then, the precipitated formazan crystals, a purple insoluble MTT product, were dissolved by 800 μL acidified isopropanol (0.2 mL of 0.04 N hydrochloric acid (HCl) in 10 mL of isopropanol). After the incubation period, the absorbance values at 540 nm were measured on a multifunctional microplate scanner (Tecan Infinite M200).

To investigate the effect of the microcarriers the on cell adhesion and spreading, the MC3T3-E1 cell on the surface of different microcarriers were visualized was visualized by a ‘Live/Dead’ assay kit only using the ‘Live’ kit (green) (Biotium, USA), and nuclei staining (blue) using 4,6-diamidino-2-phenylindole dihydrochloride (DAPI, Invitrogen). For the ‘Live/Dead’ assay kit, cell/microcarriers were first rinsed with PBS and then incubated in the standard working solution for 1 h. For nuclei staining, cell/microcarriers were fixed using 4% PFA for 15 min, followed by washing in PBS for three times. Afterwards, cells were incubated with DAPI for 3 min and then washed in PBS three times. Finally, the cell/microcarriers samples were observed under a fluorescence microscope (TE2000-U, Nikon).

### Alkaline phosphatase (ALP) activity

The components of the medium and operation specifications for cell culture were in accordance with cell proliferation assays. After incubating for 7 and 14 days on various microcarriers, cellular ALP activity was investigated via pNPP assay. Briefly, the medium of each well was carefully removed and cells were washed three times with PBS. Then, cells were lysed in RIPA buffer before freezing at −80 °C for 30 min and thawing at 37 °C. A 50 μL portion of the pNPP solution was placed in every well away from light and maintained at 37 °C for 30 min. The absorbance at 405 nm was read by a multifunction microplate scanner (Infinite M200, TECAN). Measurements were normalized by the number of cells from BCA protein assay.

### Quantitative real-time PCR analysis

The MC3T3-E1 cells cultured on the microcarriers for 7 days were also collected for an evaluation of their osteogenic related gene expression. The expression of osteogenic genes was explored via quantitative real-time polymerase chain reaction (qRT-PCR). Total RNA was extracted using TRIzol Reagent (Invitrogen) according to the manufacturer’s protocol. The total RNA concentration and purity were detected by Nanodrop Assay (Tecan M200), and the first strand of cDNA was synthesized by reverse transcriptase as described by the M-MLV manual (Promega). The expression of osteogenic markers was quantified by qPCR SYBRGreen Mix Kit (TaKaRa). The primer sequences specific for the target gene including anti-runt-related transcription factor 2(RUNX2), osteopontin(OPN) and glyceraldehyde-3-phosphate dehydrogenase(GAPDH) used for qRT-PCR are listed in Table 1. The specificities of the listed oligonucleotides were checked by BLASTN® (Basic Local Alignment Search Tool) against the mouse RefSeq RNA database at NCBI. The qPCR amplification was performed follows: initial denaturation at 95 °C for 10 min, followed by 40 cycles at 95 °C for 30 s, 58 °C for 1 min, and 72 °C for 1 min. A comparative threshold cycle method was used to analyse the Q-PCR results using iCycleriQ Detection System software with GAPDH as the reference gene. All results were quantified using the ΔΔCt relative quantification method.

**Table 1:** List of Genes and Primer Nucleotide Sequences.

Gene	Forward primer sequence	Reverse primer sequence
RUNX 2	5-GCCGGGAATGATGAGAACTA -3′	5-GGACCGTCCACTGTCACTTT -3′
OPN	5-TCAGGACAACAACGGAAAGGG -3′	5-GGAACTTGCTTGACTATCGATCAC -3′
GAPDH	5-AACTTTGGCATTGTGGAAGG -3′	5-ACACATTGGGGGTAGGAACA -3′

### Immunofluorescence Staining

For the immunofluorescence analysis, MC3T3-E1 cells were cultured on various microcarriers for 7 days at 37.8 °C in a humidified 5% CO_2_ atmosphere. The samples were permeated with 0.1% Triton-X 100 in phosphate buffer for 5 min. After being blocked using 1% BSA in phosphate buffer for 30 min, the samples were incubated with primary antibody (OPN and Runx2, 1:500, Abcam) for 60 min under ambient temperature. Then, cell/microcarriers samples were washed with PBS for three times and stained with a fluorescein isothiocyanate (FITC)-labeled secondary antibody (1:500, Abcam) for 60 min. Finally, cell nuclei were dyed with 4′,6-diamidino-2-phenylindole (DAPI). Photos were taken on a multifunctional microplate scanner (Tecan Infinite M200).

### Mineralization

Calcium deposition was determined by alizarin red S (ARS) staining of the MC3T3-E1 cells. After 20 d of culture, cell/microcarriers samples were fixed in 4% paraformaldehyde in PBS for 15 min at room temperature and then washed with acidic PBS (pH 4.2) for three times. The samples were stained with Alizarin Red S solution (50 mM) for 20 min at 37 °C. Then, ARS stained samples were washed with PBS and followed by treatment with 1 ml of 10% CPC (Cetylpyridinium chloride) solution for calcium quantification. The absorbance of the solution was read at 540 nm in a multifunctional microplate scanner. Furthermore, after 20 d of culture, the cells/microcarrier samples were washed and fixed with 4% glutaraldehyde solution for 15 min. Afterwards, samples were dehydrated in graded ethanol solutions (50, 70, 80, 90, 95 and 100%) and air-dried. Finally, the samples were coated with gold and observed by SEM.

### Statistical analysis

The data were analysed using Origin 8.0 and are presented as the mean ± standard deviation. Statistical analysis was performed using the one-way analysis of variance to determine significant differences. A value of p < 0.05 was considered statistically significant.

## Results and Discussion

### Characterization of Microcarriers

**Figure 1:**
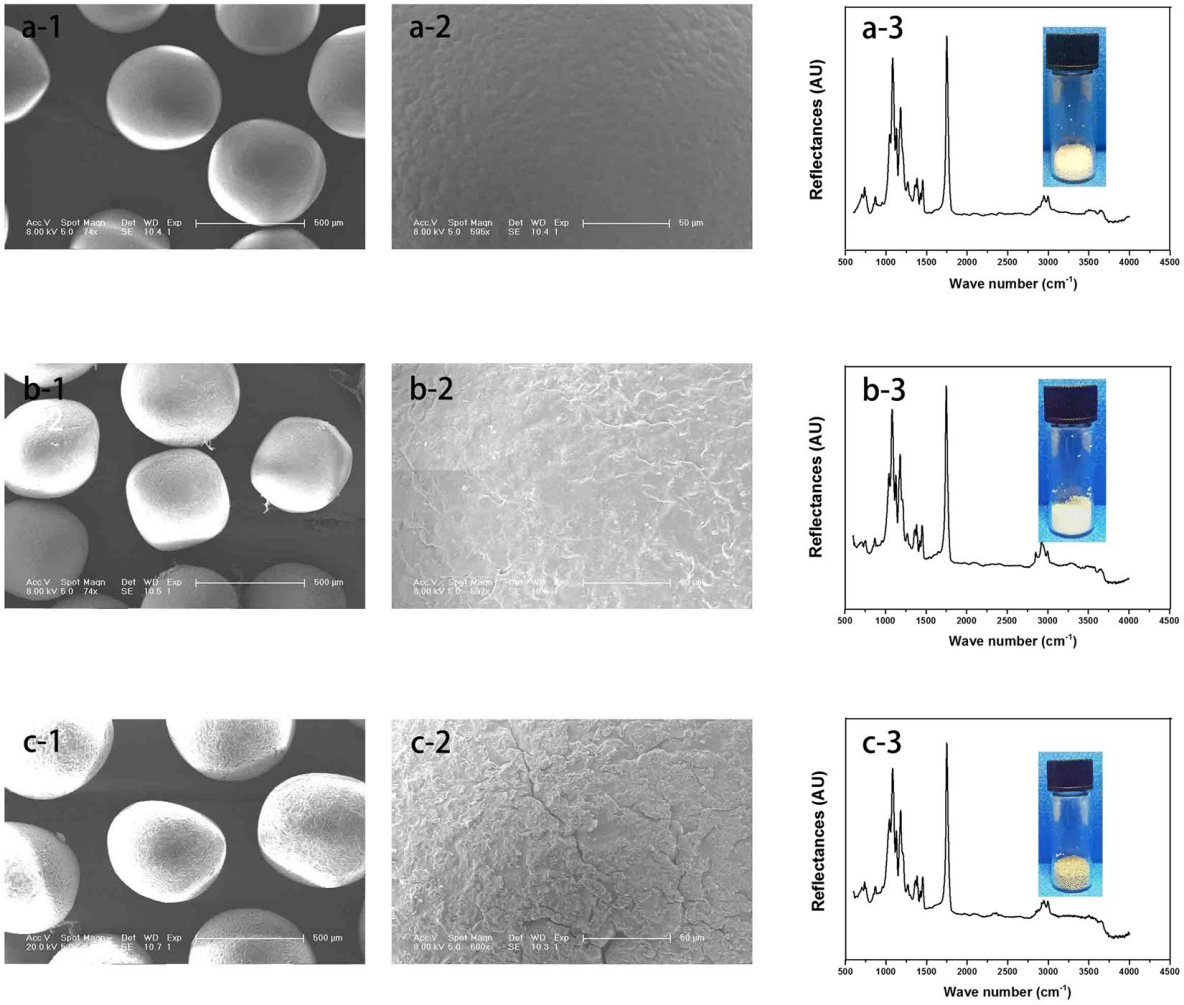
SEM images (1-2) and FTIR spectra (3) of PLGA (**a**), PLGA/HA (**b**) and GO-PLGA/HA (**c**) microcarriers. Bar lengths are 500 μm (a-1, b-1, c-1, and d-1) and 50 μm (a-2, b-2, and d-2).

In this study, we focused on the preparation of BMP-2 immobilized graphene oxide-incorporated PLGA/HA microcarriers to improve cell adhesion and osteogenic activity on bone implants for osseointegration. GO-PLGA/HA microcarriers were prepared on the basis of the admixture of PLGA and HA blended with GO by an emulsion solvent evaporation method. The microscopic morphology and macroscopic appearance of the PLGA, PLGA/HA and GO-PLGA/HA microcarriers were shown in Fig. 1. As shown in Fig. 1(c), the GO-PLGA/HA microcarriers can be easily distinguished by the colour change of the microcarriers (brown) due to being impregnated GO. The surface morphology of various microcarriers was also observed by SEM. It could be seen from Fig. 1(a) that pure PLGA microcarriers were almost spherical and had a smooth surface. After blending with HA, the surface of PLGA/HA mcrocarriers was rougher compared with the surface of PLGA microcarriers. Furthermore, blending of PLGA/HA with GO caused an increase in the surface roughness of microcarriers while the spherical form of the microcarriers was retained. The average diameter of the microcarriers was 521.11 ± 60.21 μm and the average diameters of the three different microcarriers do not show significant differences.

**Figure 2:**
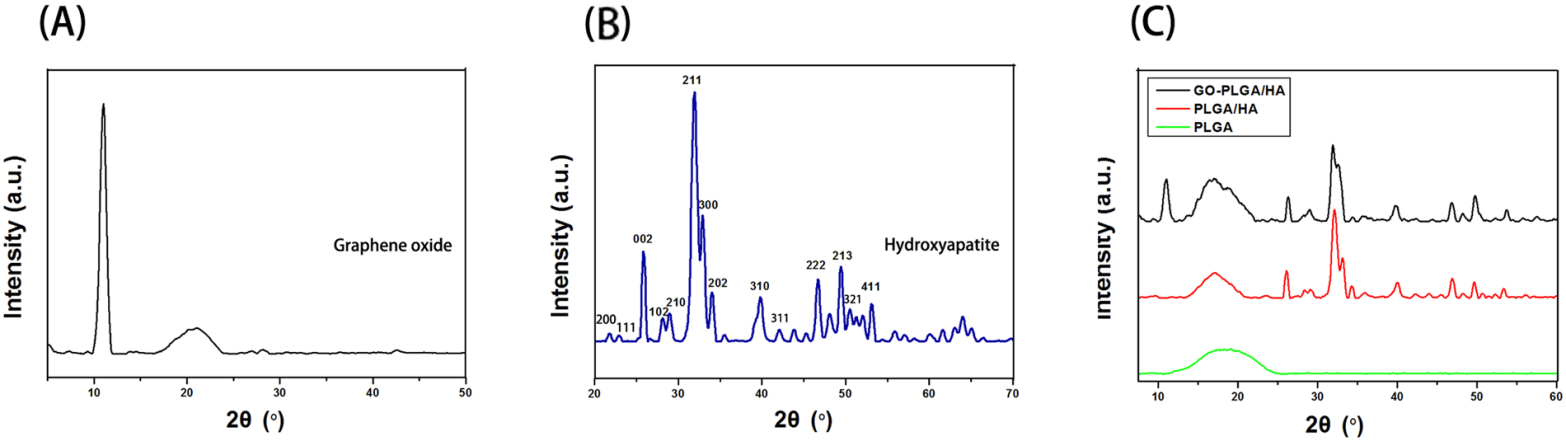
XRD patterns of GO (**A**), HA (**B**), and microcarriers (**C**) of PLGA, PLGA/HA and GO-PLGA/HA.

**Figure 3:**
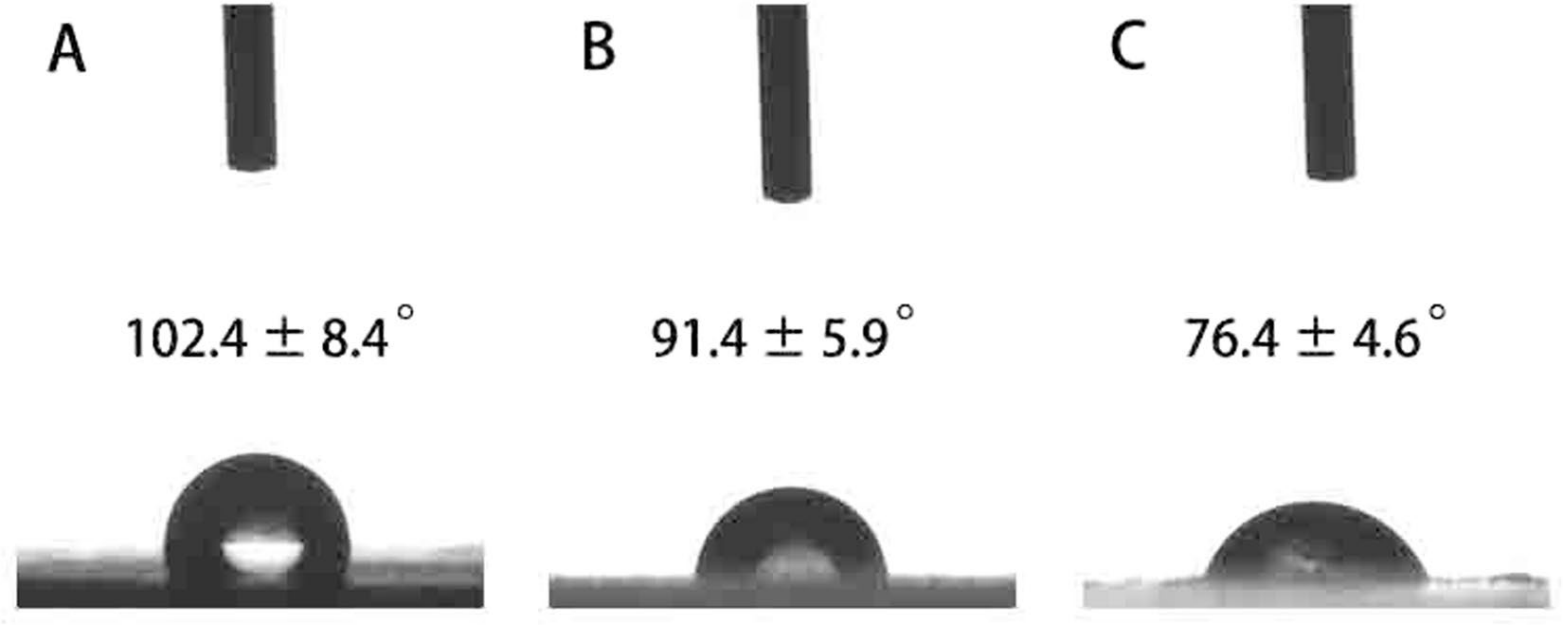
Water contact angles of pure PLGA microcarriers (**A**), PLGA/HA microcarriers (**B**) and GO-PLGA/HA microcarriers (**C**).

The FTIR spectra of pure PLGA, PLGA/HA and GO-PLGA/HA microcarriers were performed to determine the surface chemical properties of microcarries. As shown in Fig. 1(a-3), the peaks at 2990 and 2940 cm^−1^(–CH_3_), 1753 cm^−1^(C=O), 1183 and 1083 cm^−1^(C-O), correspond to the PLGA. The existence of HA in the PLGA microcarriers is confirmed by the appearance of a peak at 500–600 cm^−1^, which is ascribed to the PO_4_^3−^ characteristic peaks. Compared with the PLGA/HA microcarriers, the FTIR spectra of GO-PLGA/HA microcarriers showed no visible alteration, indicating that the GO dispersed into the PLGA only by physical mixing instead of a chemical reaction^33^. The XRD patterns of GO, HA and mcrocarriers are presented in Fig. 2. The principal diffraction peaks for the typical HA crystalline planes (200), (111), (002), (102), (210), (211), (300), (202), (310), (311), (222), (213), (321) and (411) are found in the XRD patterns of PLGA/HA and GO-PLGA/HA microcarriers. Furthermore, as shown in Fig. 2, the GO pattern shows a characteristic peak at 2θ≈11°, corresponding to an interlayer spacing of 0.79 nm, which is the typical separation of the layered GO. The XRD patterns of GO-PLGA/HA mcrocarriers exhibited a similar diffraction peak at 2θ≈11°. The above results suggested that there were the HA and GO were exposed on the microcarrier surface.

### Contact angle analysis

The hydrophilicity of the materials plays an important role in interacting with cells. In addition, the ability to delivery growth factors across the interface of the biomaterials with biological factors also depends on the hydrophilicity of the biomaterials. We measured the water contact angle of PLGA, PLGA/HA and GO-PLGA/HA films surfaces to analyse the change in wetting of the microcarriers surface. As shown in Fig. 3, the contact angle on pure PLGA films was 102.4 ± 8.4° for the PLGA, and 91.4 ± 5.9° for the PLGA/HA films due to the exposure of HA on the surface. After blending with GO, the average water contact angle dramatically decreased from 91.4 ± 5.9° for the PLGA/HA films to 76.4 ± 4.6° for the GO-PLGA/HA films, indicating a significant increase of wettability due to the blending the substrates with GO. Our results demonstrated that the hydrophobicity of the PLGA/HA microcarriers was slightly reduced after the GO incorporation.

### Protein Adsorption Studies

**Figure 4:**
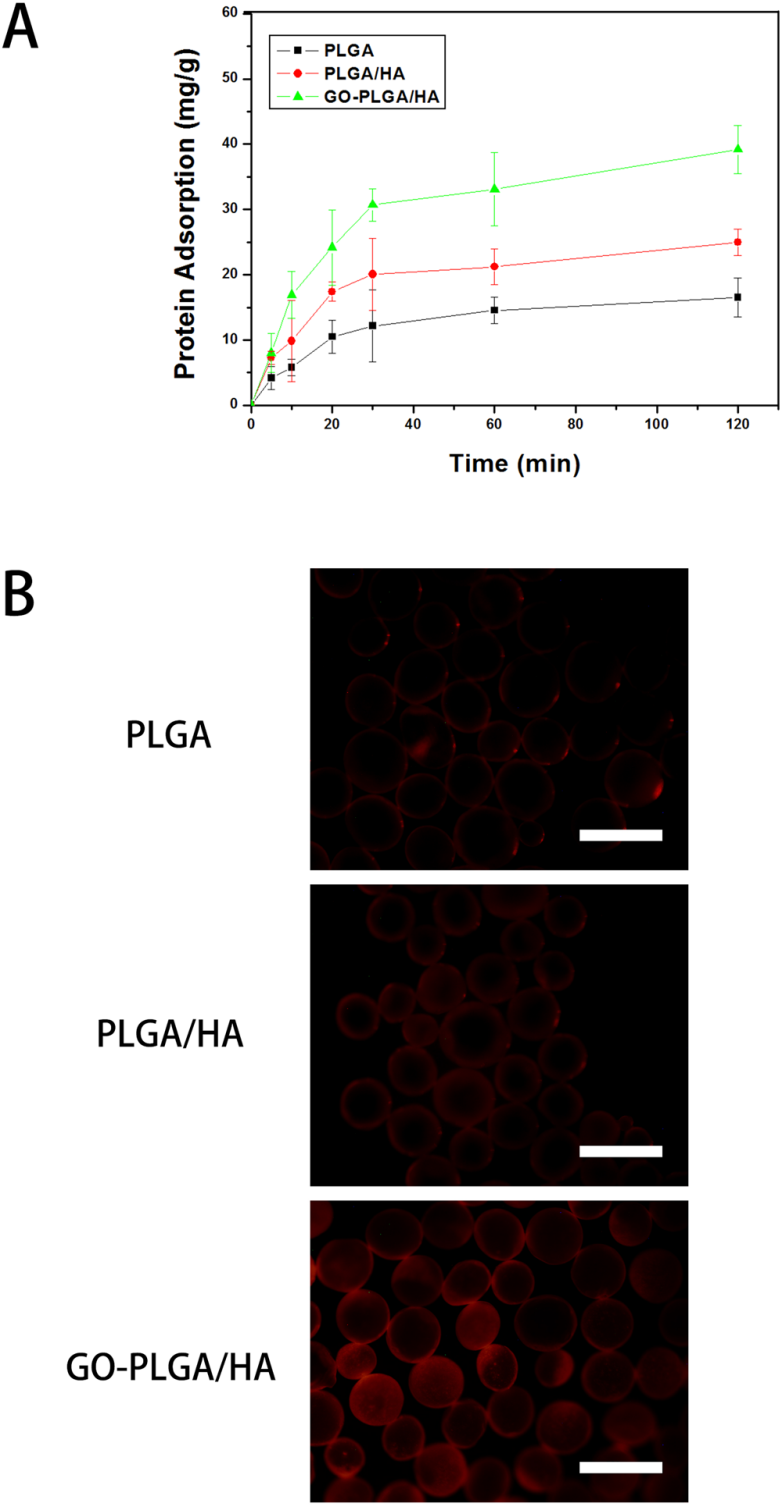
Adsorption isotherms of BSA over PLGA, PLGA/HA and GO-PLGA/HA microcarriers (**A**). Fluorescence images of the Rhodamine B-BSA adsorption on PLGA, PLGA/HA and GO-PLGA/HA microcarriers (**B**). The scale bar was 500 μm, error bars represent standard deviation for n = 3.

The protein adsorption studies of BSA as a model protein onto each kind of microcarriers are shown in Fig. 4. After incubation for 2 h, it could be seen that the BSA adsorption on PLGA/HA and GO-PLGA/HA microcarriers was obviously higher than that those of pure PLGA microcarriers. PLGA microcarriers showed the lowest protein adsorption capacity for its weak interactions between BSA molecules. Moreover, the BSA adsorption capacities of PLGA/HA microcarriers were obviously higher than that of PLGA microcarriers. It is deduced that the higher protein adsorption capacities of PLGA/HA microcarriers might result from their improved surface properties and larger specific surface areas after the incorporation of HA crystals. Among microcarriers, GO-PLGA/HA microcarriers have the strongest ability of protein adsorption. It was reported that GO has strong capabilities to adsorb various proteins, including cytochrome c, bovine serum albumin, ribonuclease A, and protein kinase A^34–36^. Furthermore, compared to PLGA/HA microcarriers, the GO-PLGA/HA surface showed a rougher surface morphology and exhibited a higher protein adsorption. This also implies the important relationship between protein adsorption and surface morphology. On the other hand, the improvement of hydrophilicity of the GO-PLGA/HA microcarriers can also improve the affinity of microcarriers for proteins, which would be favourable for more protein to be immobilized on the surface of microcarriers. The above results suggested that GO-PLGA/HA microcarriers have good affinity with protein and may be an effective carrier for BMP-2 delivery.

### Cell adhesion and proliferation

**Figure 5:**
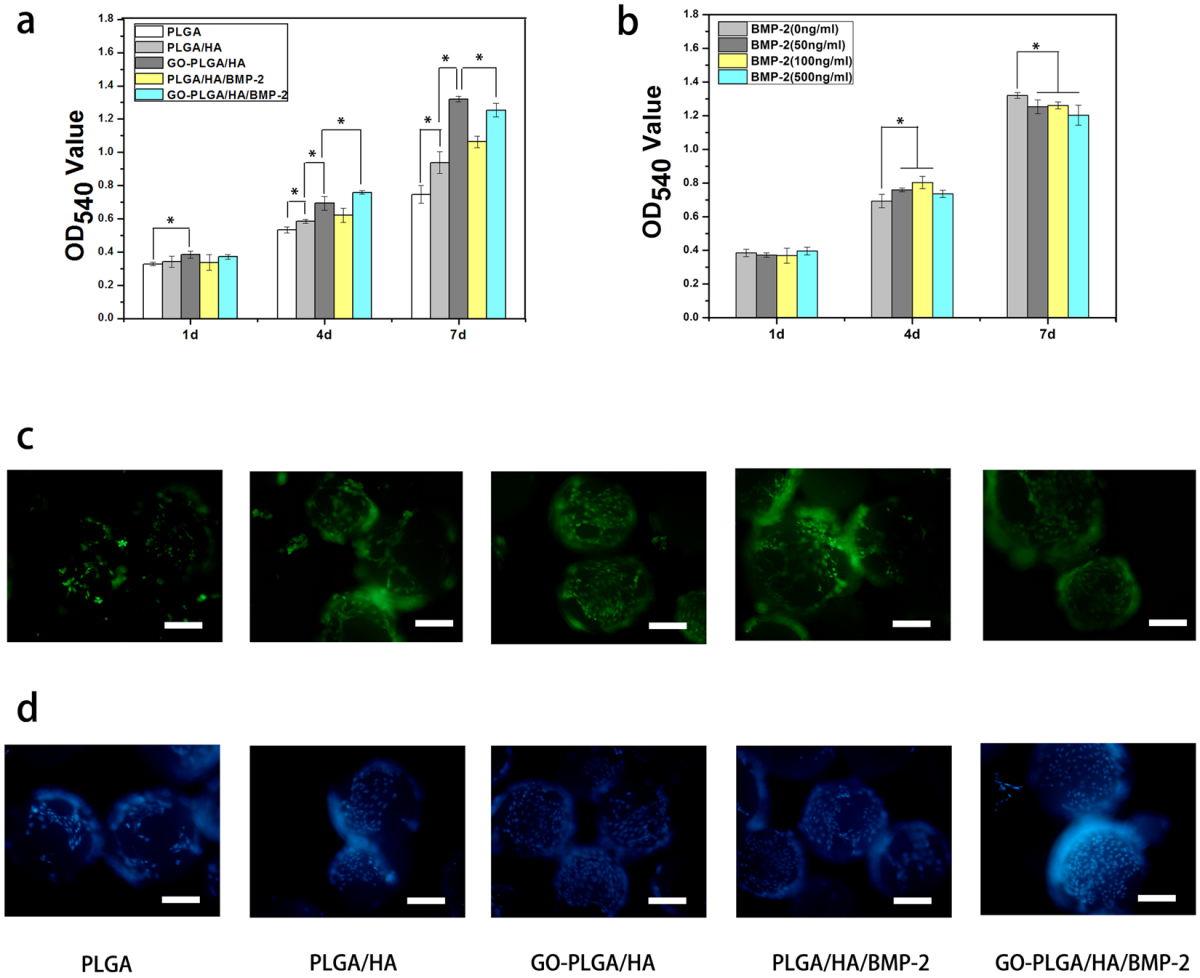
(**a**) Proliferation of MC3T3-E1 cells in PLGA, PLGA/HA, GO-PLGA/HA, PLGA/HA/BMP-2 and GO-PLGA/HA/BMP-2 microcarriers by MTT. (**b**) Proliferation of MC3T3-E1 cells in different microcarriers immobilized with BMP-2. The concentrations of BMP-2 in the immobilized solution were 0, 50, 100 and 500 ng·mL^−1^. (**c**) Live-dead and (**d**) DAPI staining of MC3T3-E1 cells cultured in PLGA, PLGA/HA, GO-PLGA/HA, PLGA/HA/BMP-2 and GO-PLGA/HA/BMP-2 microcarriers at day 4. The scale bar was 200 μm. P < 0.05, n = 4.

The enhancement of cell adhesion and proliferation on the bone scaffolds is usually responsible for eventual tissue integration and the amount of new bone formation. Accordingly, the cell adhesion and proliferation on the microcarriers are important factors to evaluate microcarriers as injectable scaffolds for the application of bone defect repair. The proliferation of MC3T3-E1 cells on the different microcarriers (PLGA, PLGA/HA, GO-PLGA/HA, PLGA/HA/BMP-2 and GO-PLGA/HA/BMP-2) was assessed using an MTT assay from 1 to 7 d. As shown in Fig. 5(a), the proliferation of MC3T3-E1 cells was observed on all microcarriers with increasing incubation up to 7 days. After 4 and 7d of culture, the OD values of cell viability was slightly increased in the PLGA/HA microcarriers compared with pure PLGA microcarriers (p < 0.05). Previous studies demonstrated that HA could enhances the attachment, proliferation, and even osteogenesis differentiation of cell on PLGA scaffold, due to its rough surface and good biocompatibility^15,37,38^. After GO was added to the PLGA/HA microcarriers, the cells exhibited more apparent viability in the GO-PLGA/HA microcarriers compared with the PLGA and PLGA/HA mcrocarriers (p < 0.05). The excellent cell proliferation on the GO-PLGA/HA microcarriers might be attributed to the rapid absorption of protein due to the π–π stacking between aromatic rings in the GO, thus providing a biocompatible environment for cells to adhere and proliferate that ultimately attracts more cells to adhere on the microcarriers. The surface properties of microcarriers are also play an important role in the growth, adhesion and migration of cells. It is speculated that that the improved cell viability on the GO-PLGA/HA microcarriers could be attributed to the higher roughness on the surface of GO-PLGA/HA microcarriers. Furthermore, hydrophilicity as a main characteristic of biomaterials has a significant impact on cell attachment and growth. Therefore, the improved hydrophilicity of GO-PLGA/HA microcarriers is also a key reason for higher cell viability.

Despite improving the cell adhesion and proliferation of the microcarriers, BMP-2 was immobilized on the surface of microcarriers to make the microcarriers become more osteoinductive. After 4 d of culture, a higher OD value was found on the BMP-2-functionalized microcarriers compared with the pristine PLGA/HA and GO-PLGA/HA microcarriers. Among the five groups of microcarriers, GO-PLGA/HA/BMP-2 demonstrated the highest ability in promoting cell proliferation (p < 0.05). However, after 7 d of culture, the OD values of MC3T3-E1 cells on all the GO-PLGA/HA/BMP-2 microcarriers were observed lower than the GO-PLGA/HA microcarriers. In addition, it was found that the BMP-2-immobilized microcarriers with a high concentration have a lower OD value after 7 days of culture. As cells proliferated to a certain number, the proliferation stopped and turned into osteogenesis differentiation^39^. Therefore, we speculated that the lower OD values of MC3T3-E1 cells on GO-PLGA/HA/BMP-2 microcarriers represented the beginning of the cell osteogenesis differentiation.

We examine the effect of the HA, GO and immobilized BMP-2 on the adhesion of MC3T3-E1 cells. As shown in Fig. 5d, the nuclear staining results indicated that the number of MC3T3-E1 cells that grew on PLGA/HA and GO-PLGA/HA microcarriers was more than the PLGA microcarriers. Compared to the PLGA/HA,microcarriers, more adhered MC3T3-E1 cells were observed on GO-PLGA/HA microcarriers, indicating that the combination of HA and GO can improve the biocompatibility of the microcarriers and thus induces better cell growth. A live/dead assay was used to determine the cell attachment and viability on the materials. (Fig. 5c). Live cells were stained green and exhibited a normal polygonal morphology. Compared to the PLGA microcarriers, more live cells were found on the surface of PLGA/HA and GO-PLGA/HA microcarriers. Furthermore, there were greater cell quantities and positive cellular interactions on BMP-2 immobilized microcarriers. On the other hand, it was found that BMP-2 immobilized using GO-PLGA/HA microcarriers promoted greater cell attachment and proliferation than BMP-2 immobilized using PLGA/HA microcarriers *in vitro*. Thus, these results indicated that BMP-2 immobilization GO-PLGA/HA microcarriers can provide an appropriate microenvironment for the attachment and proliferation of cells.

### ALP activity

**Figure 6:**
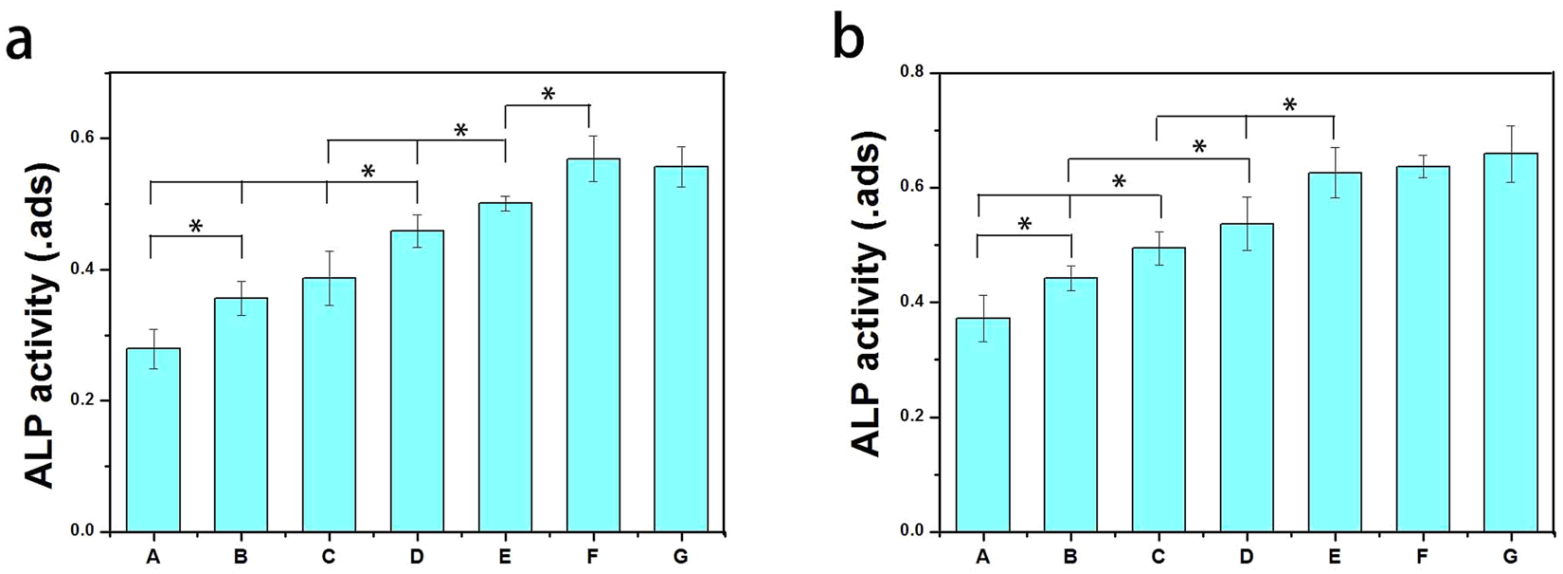
ALP activities of MC3T3-E1 cells on different microcarriers for 7 d (**a**) and 14 d (**b**) analyzed with pNPP kit: PLGA (A); PLGA/HA (B); GO-PLGA/HA (C), PLGA/HA/BMP-2(50 ng·mL^−1^) (D) and GO-PLGA/HA/BMP-2 (50, 100 and 500 ng· mL^−1^) (E-G). p < 0.05, n = 4.

ALP enzyme activity, as a marker of early osteogenic differentiation, was chosen to explore the osteoinductive activity of the different microcarriers. The ALP activity of MC3T3-E1 cells cultured on different microcarriers were measured at 7 and 14 days. As shown in Fig. 6, there was higher ALP activity on the GO-PLGA/HA microcarriers than on the PLGA/HA microcarriers at both 7 and 14 d. The intrinsic properties of graphene are thought to increase cytoskeleton tension, thus guiding cell behavior such as osteogenesis differentiation^40^. Furthermore, due to hydrogen bonding and electrostatic interactions, graphene and its sderivatives allows the non-covalent binding of proteins and osteogenic inducers on its surface^41^. Previous studies have demonstrated that GO could significantly increase ALP expression of a variety of cells^33,42^. Furthermore, at 7 and 14 days, the ALP activity of MC3T3-E1 cells in the BMP-2-modified microcarriers was higher than those of cells on the PLGA, PLGA/HA and GO-PLGA/HA microcarriers (p < 0.05), suggesting that the osteogenic differentiation of cells was better on the BMP-2-modified microcarriers than other microcarriers. An obvious rise in the ALP activity of MC3T3-E1 cells was noticed on the GO-PLGA/HA/BMP-2 microcarriers, being superior to the samples of PLGA/HA/BMP-2 microcarriers at both 7 and 14 d (p < 0.05). It was deduced that the high protein affinity of GO-PLGA/HA microcarriers cause a higher immobilized efficiency of BMP-2 than PLGA/HA microcarriers, and the immobilized BMP-2 could retain its bioactivity for a longer time and make the microcarriers perform long-term osteoconductivity. To better investigate the osteoinductive effect of immobilized BMP-2, the *in vitro* osteogenic differentiation efficacy of the microcarriers treated with different concentration of BMP-2 was also investigated. Fig. 6 showed that higher ALP activity was found in the microcarriers treated with medium and high concentrations of BMP-2 at 7 d and 14 d. Among the BMP-2-modified microcarriers, as low as 100 ng·mL^−1^ of immobilized BMP-2 was most effective in the enhancement of cell early osteogenic differentiation in this study.

### Mineralization

**Figure 7:**
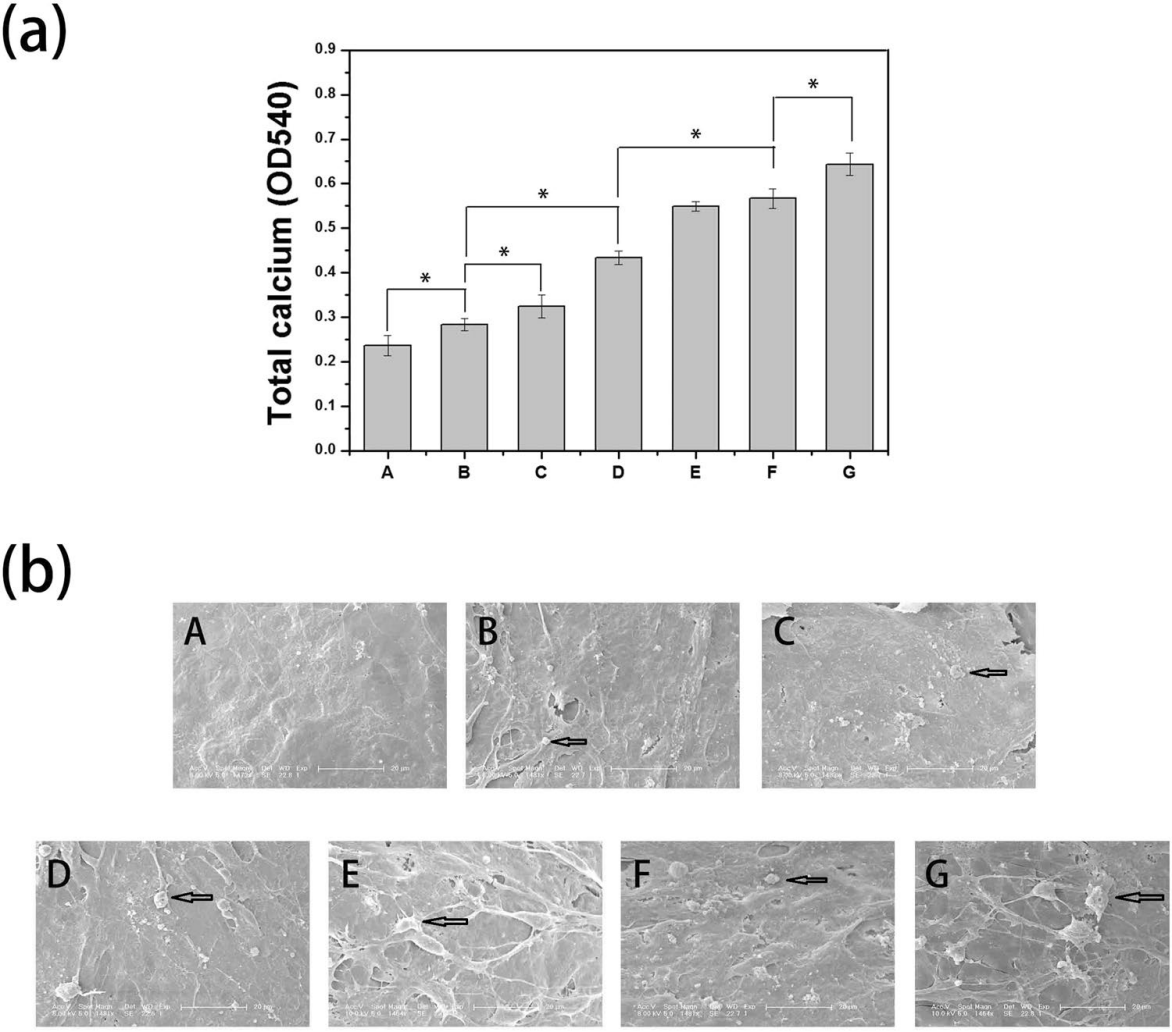
(**a**) The corresponding quantitative evaluation of calcium content mineral deposition in MC3T3-E1 cells cultured for 20 d. (**b**) SEM images of MC3T3-E1 cells on different microcarriers surface. (A) PLGA, (B) PLGA/HA, (C) GO-PLGA/HA, (D) PLGA/HA/BMP-2(50 ng · mL^−1^), (E-G) GO-PLGA/HA/BMP-2 (50, 100 and 500 ng·mL^−1^). All scale bar lengths are 200 μm. P < 0.05, n = 4.

The capacity of mineral deposition reflects osteogenesis and has been regarded as a marker for bone regeneration. In this study, the assessment of quantitative cell mineralization was performed by extracting Alizarin Red with 10% cetylpyridinium chloride (CPC), which was employed to determine calcium mineralization on the microcarriers. As shown in Fig. 7, after 20 days of culture, the calcium content in MC3T3-E1 cells on GO-PLGA/HA was significantly higher than that of cells growing on PLGA/HA microcarriers. The incorporation of GO nanosheets can effectively facilitates calcium deposition in MC3T3-E1 cells. We speculated that the excellent protein adsorption and hydrophilicity of GO could not only promote cell proliferation but also improve the nucleation of HA, which facilitated the late stage marker of osteogenic differentiation. After the microcarriers incorporating BMP-2, all BMP-2-modified microcarriers showed higher calcium content than other groups, which implied that BMP-2 played an importance role in promoting the osteogenic differentiation of MC3T3-E1 cells. In accordance with the ALP results, the highest calcium content was observed over the GO-PLGA/HA/BMP-2 microcarriers. Furthermore, we found that the high concentration of BMP-2 could better induce MC3T3-E1 cell mineralization during the later stage of differentiation. The above results further conformed that BMP-2-immobilized GO-PLGA/HA microcarriers promote osteogenic differentiation and enhance the metabolic activity of osteoblasts.

To better observe the effect of different microcarriers on cell mineralization, the calcium deposition of MC3T3-E1 cells was also observed through SEM as evidence for MC3T3-E1 cells osteogenic differentiation. The SEM images (Fig. 7b) showed that more apatite particles (black mark) on GO-PLGA/HA microcarriers were found over pure PLGA and PLGA/HA microcarriers. Compared to the pristine PLGA/HA and GO-PLGA/HA microcarriers, the cells grown on the surface of BMP-2-modified microcarriers had increased mineralized nodule formation. The most densely apatite particles was observed over the GO-PLGA/HA/BMP-2 microcarriers. The SEM results also depicted the same trends observed from the quantitative assessment of mineral deposition, demonstrating that the BMP-2 immobilized GO-PLGA/HA microcarriers can significantly enhance the osteodifferentiation of MC3T3-E1 cells.

### Bone-Related Gene Expression by qRT-PCR Tests

**Figure 8:**
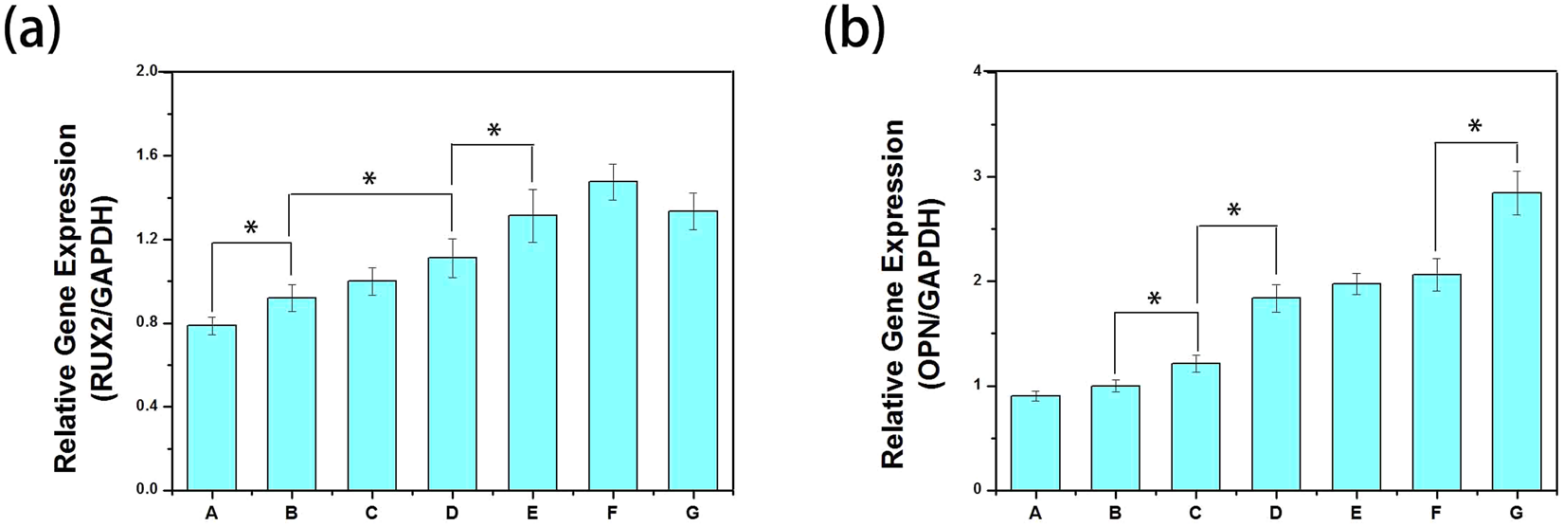
Quantitative real-time PCR analysis of osteogenesis-related gene expression of Runx2 (**a**) and OPN (**b**) after MC3T3-E1 cells cultured for 7d: PLGA (**A**); PLGA/HA (**B**); GO-PLGA/HA (**C**); PLGA/HA/BMP-2 (50 ng·mL^−1^) (**D**); GO-PLGA/HA/BMP-2 (50, 100 and 500 ng·mL^−1^) (**E**–**G**). P < 0.05, n = 3.

The main characteristics of osteogenesis differentiation are often accompanied by the up-regulation or down-regulation of certain genes in each stage. For example, Runx2 is an early osteogenesis differentiation marker observed at the early stage of differentiation, while OPN expression is observed at the middle/later stage of differentiation. The osteogenic gene expression of MC3T3-E1 cells cultured on different microcarriers for 7 days was analysed using quantitative real-time PCR. As shown in Fig. 8, both Runx2 and OPN expression were slightly higher for GO-PLGA/HA than for the PLGA/HA microcarriers at 7 days, which is indicated that the GO combined with HA could enhanced the osteoinductivity of microcarriers. After BMP-2 immobilization, BMP-2-modified microcarriers showed higher expression levels of Runx2 and OPN than those of pristine microcarriers, indicating much stronger osteogenic induction by BMP-2. Compared to PLGA/HA/BMP-2 microcarriers, there were higher increase in Runx2 expression on GO-PLGA/HA/BMP-2 microcarriers, and the highest level of Runx2 expression were found in the microcarrier treated with a medium concentration of BMP-2. Furthermore, slightly higher OPN gene expression at 7 days on PLGA/HA/BMP-2, GO-PLGA/HA/BMP-2 (50 ng mL^−1^), and GO-PLGA/HA/BMP-2 (100 ng·mL^−1^) groups was observed without a significant difference, but the gene expression of OPN was significantly promoted by the microcarriers treated with a high concentration of BMP-2. We speculated that the medium concentration of BMP-2 was more effective in the enhancement of cell osteogenic differentiation at the early stage of differentiation. However, the high concentration of BMP-2 had a greater impact on the promotion of cell osteogenic differentiation at the middle/later stage of differentiation than the low/medium concentration of BMP-2.

### Bone-Related Protein Expression by Immunofluorescence Staining

**Figure 9:**
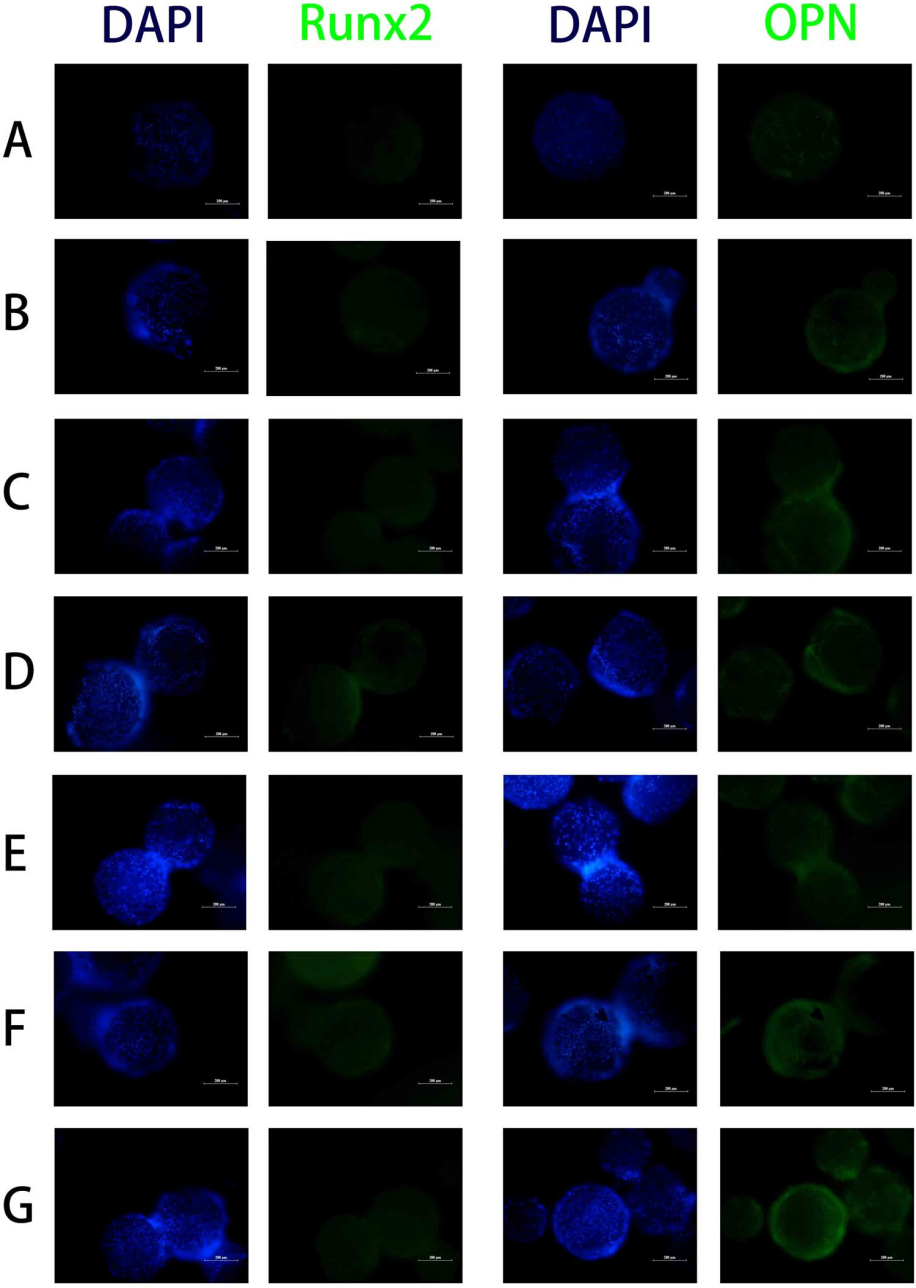
*In vitro* Runx2 and OPN protein expression of MC3T3-E1 cells cultured on different microcarriers for 7d: PLGA (**A**); PLGA/HA (**B**); GO-PLGA/HA (**C**); PLGA/HA/BMP-2 (50 ng·mL^−1^) (**D**); GO-PLGA/HA/BMP-2 (50, 100 and 500 ng·mL^−1^) (**E**-**G**). All scale bar lengths are 200 μm.

To better observe the effect of different microcarriers on cell osteogenesis differentiation, the protein expression of Runx2 and OPN by the MC3T3-E1 cells grown on different microcarriers was evaluated by immunofluorescence staining. As shown in Fig. 9, after 7 days of culture, the Runx2 and OPN protein expression in the GO-PLGA/HA microcarriers were higher than those in the PLGA/HA microcarriers. After BMP-2 immobilization, there was a higher expression level of Runx2 and OPN protein in the BMP-2 treated group than in the pristine group at 7d. Furthermore, the highest Runx2 and OPN expressions were observed on the GO-PLGA/HA/BMP-2 (100 ng mL^−1^) and GO-PLGA/HA/BMP-2 (500 ng·mL^−1^) microcarriers, respectively. The protein expression results were well in accordance with the observation of ALP activity, calcium deposits, and bone-related gene expression.

So far biodegradable microcarriers have been developed for bone tissue engineering. An important advantage of the biodegradable microcarriers is that can provide a sufficient number of anchorage sites and better facilitate cell attachment^5,6,10^. HA has good biocompatibility and osteoconductivity, and the superior biodegradability of PLGA and HA nanocomposites in the form of bone grafts has been reported in previous studies. Recently, much attention has also been paid to the fabrication of PLGA/HA microcarriers^37,43^. Furthermore, active factors, such as bone morphogenetic protein-2 (BMP-2), could make the biodegradable microcarriers possess osteogenesis induction activity. However, the poor hydrophilicity and lack of functional groups of the polymers often result in lower growth factor loading efficiency. Therefore, the GO was employed in this study for pre-modification of the PLGA/HA microcarriers, which can improve the bioactivity of the PLGA/HA microcarriers and efficient immobilization of BMP-2 onto the surface of microcarriers. More recent reports reveal that the GO can effectively enhance surface properties of polymer materials due to its unique chemical structures composed of small sp2 carbon domains surrounded by sp3 carbon domains and oxygen-containing hydrophilic functional groups^42,44^. In this study, the protein adsorption and hydrophilicity of the PLGA/HA microcarriers were increased by the incorporation of GO due to improved surface properties, which can provide more favourable microenvironments for cell adhesion and growth. Another advantage of GO is that the improved protein adsorption capacity of microcarriers can effectively enhance BMP-2 binding on the surface of the microcarriers, which make the microcarriers perform long-term osteoconductivity. The π-electron clouds of GO are capable of interacting with the inner hydrophobic cores of BMP-2 protein. Furthermore, the negatively charged COO– domains of GO-COO– can also bind with positively charged BMP-2 through electrostatic interactions^45,46^. These interactions are thought to be responsible for the sustained release of BMP-2 from GO. Our *in vitro* data showed that the attachment and proliferation of MC3T3-E1 cells were increased obviously by the incorporation of GO. After BMP-2 immobilized, the ALP activity, calcium deposition and bone-related gene expression of MC3T3-E1 cells on GO-PLGA/HA/BMP-2 microcarriers was obviously stronger than PLGA/HA/BMP-2 microcarriers. The enhanced *in vitro* osteogenic activity of BMP-2 in the GO-PLGA/HA microcarriers may be due to the sustained release, higher stability, and higher bioactivity of BMP-2 delivered by GO-PLGA/HA microcarriers. Previous studies have demonstrated that BMP-2 adsorbed on GO was protected from protein denaturation and was released in a bioactive form^47,48^. Therefore, we can speculate that the use of BMP-2-immobilized GO-PLGA/HA microcarriers was an effective option for the regeneration of bone defects. Meanwhile, an *in vivo* study of the biodegradable microcarriers is necessary to achieve more conclusive outcomes for bone defect therapy in our future work.

## Conclusions

In summary, the biodegradable GO-PLGA/HA microcarriers have been successfully fabricated by an emulsion solvent evaporation method. We found that the infusion of GO could effectively enhance surface properties of PLGA/HA microcarriers, and the adhesion, proliferation and osteogenesis differentiation of MC3T3-E1 cells were greatly enhanced on the GO-PLGA/HA microcarriers. Furthermore, the introduction of GO on the PLGA/HA microcarriers could increase the binding sites of the PLGA/HA microcarriers to BMP-2, which could effectively enhance BMP-2 binding on the surface of the microcarriers. After immobilized BMP-2, the GO-PLGA/HA microcarriers exhibited excellent bioactivities for supporting the adhesion, proliferation, and osteogenic differentiation of MC3T3-E1 cells. The immobilization of BMP-2 via GO not only decreased the growth factor consumption, but also conferred a long-term osteoinductive effect. These results indicated that GO-PLGA/HA microcarrier might be an effective carrier for BMP-2 delivery, and the BMP-2 immobilized GO-PLGA/HA microcarriers should be an excellent bone grafts for bone defect repair.

## Electronic supplementary material

Dataset 1
