# Author Correction: Enhancing Cell Proliferation and Osteogenic Differentiation of MC3T3-E1 Pre-osteoblasts by BMP-2 Delivery in Graphene Oxide-Incorporated PLGA/HA Biodegradable Microcarriers

**DOI:** 10.1038/s41598-020-63110-8

**Published:** 2020-04-07

**Authors:** Chuan Fu, Xiaoyu Yang, Shulian Tan, Liangsong Song

**Affiliations:** 1 Department of Hand and Foot surgery, The First Hospital of Jilin University, Xinmin Street No. 71, Changchun, TX 130021 P.R. China; 2 The First Hospital and Institute of Immunology, the First Hospital of Jilin University, Xinmin Street No. 71, Changchun, TX 130021 P.R. China; 3 Department of Orthopedic Surgery, the Second Hospital of Jilin University, Ziqiang Street No. 218, Changchun, TX 130041 P.R. China

Correction to: *Scientific Reports* 10.1038/s41598-017-12935-x, published online 02 October 2017

This Article contains an error in Figure 7. In Figure 7b the SEM images of MC3T3-E1 cells within panels C and F were incorrect. The correct Figure 7 appears below as Figure 1.

**Figure 1:**
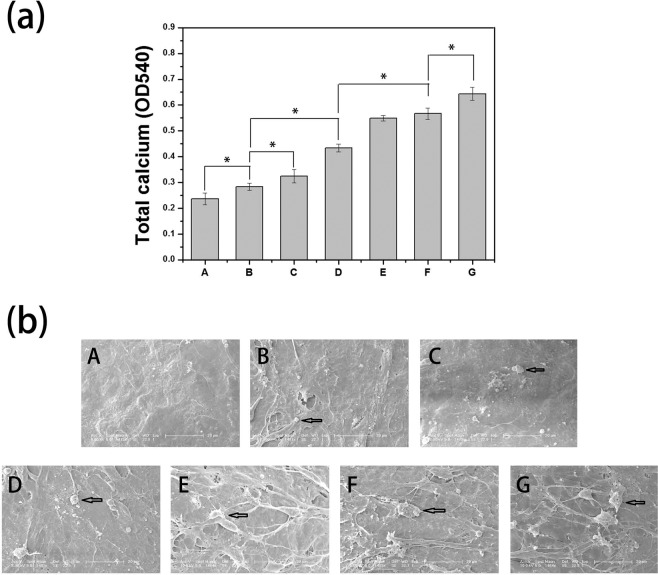
.

