# Supplementary material for: Enhancing Cell Proliferation and Osteogenic Differentiation of MC3T3-E1 Pre-osteoblasts by BMP-2 Delivery in Graphene Oxide-Incorporated PLGA/HA Biodegradable Microcarriers
Source: Sci Rep. 2017 Oct 2;7:12549. doi: 10.1038/s41598-017-12935-x (PMC5624967; doi:10.1038/s41598-017-12935-x)
Supplement: Supplementary file 1 — Dataset 1 [file 41598_2017_12935_MOESM1_ESM.doc]

**Support information for**

**Enhancing Cell Proliferation and Osteogenic Differentiation of MC3T3-E1 Pre-osteoblasts by BMP-2 Delivery in Graphene Oxide-Incorporated PLGA/HA Biodegradable Microcarriers**

Chuan Fu^1，3^, Xiaoyu Yang^3^, Shulian Tan^2*^, Liangsong Song^1*^

1 Department of Hand and Foot surgery, The First Hospital of Jilin University, Xinmin Street No. 71, Changchun TX: 130021, PR China

2 The First Hospital and Institute of Immunology, the First Hospital of Jilin University, Xinmin Street No. 71, Changchun TX: 130021, PR China

3 Department of Orthopedic Surgery, the Second Hospital of Jilin University, Ziqiang Street No. 218, Changchun TX: 130041, PR China

Current Address: Department of Hand and Foot surgery, The First Hospital of Jilin University, Xinmin Street No. 71, Changchun TX: 130021, PR China

*Corresponding Author

E-mail: slsjldxdyyy@163.com(SLS);

E-mail: yidishui7200@163.com (TSL);

**Supplementary Figures**

**Fig. S1** Binding efficiency of BMP-2 to PLGA/HA and GO-PLGA/HA microcarriers in different immobilized solution (BMP-2 concentration: 50, 100 and 500 ng ·mL^-1^). (A) PLGA/HA microcarriers (50 ng·mL-1). (B) GO-PLGA/HA microcarriers (50 ng·mL^-1^), (B) GO-PLGA/HA microcarriers (100 ng·mL^-1^) and (B) GO-PLGA/HA microcarriers (500 ng·mL-1). P < 0.05, n =4.


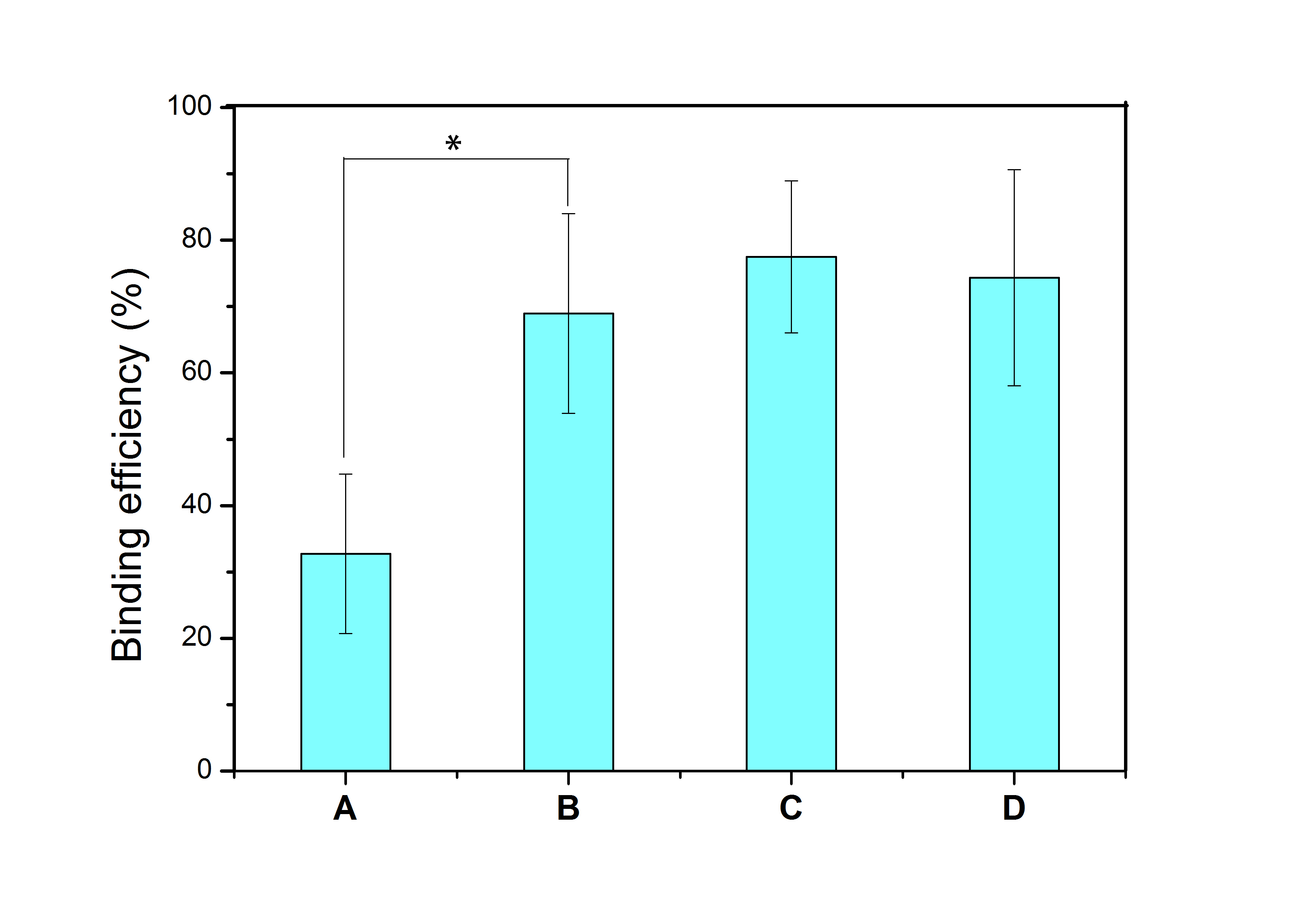


Fig. S1 Binding efficiency of BMP-2 to PLGA/HA and GO-PLGA/HA microcarriers in different immobilized solution (BMP-2 concentration: 50, 100 and 500 ng ·mL^-1^). (A) PLGA/HA microcarriers (50 ng·mL-1). (B) GO-PLGA/HA microcarriers (50 ng·mL^-1^), (B) GO-PLGA/HA microcarriers (100 ng·mL^-1^) and (B) GO-PLGA/HA microcarriers (500 ng·mL-1). P < 0.05, n =4.

**Supplementary Methods**

Determination of bound BMP-2

The microcarriers (PLGA/HA and GO-PLGA/HA) were placed into 24-well plate (10mg·well^-1^). 1 mL BMP-2 solution (the concentrations of BMP-2 in the solution were 50, 100, and 500 ng·mL^-1^) in pH 7.4 phosphate buffer saline (PBS) was added into each well. The microcarriers were incubated in the BMP-2 solution for 2 h at room temperature on a shaker. The supernatants were collected respectively. Then BMP-2 immobilized microcarriers were washed with PBS for two times. All the washing liquid were also collected and mixed with previous supernatants respectively. The amount of BMP-2 in the collected mixed solution was evaluated with an enzyme-linked immunosorbent assay (ELISA) kit according to the manufacturer’s instructions using a microplate reader (Tecan Infinite M200). Binding efficiency of BMP-2 to the different microcarriers was evaluated according to the following formula:

Binding efficiency (%) = [ ( W_a_ - W_b_ )/W_a_ ] × 100

where W_a_ and W_b_ are weight of BMP-2 respectively in PBS solution before and after incubation of the different microcarriers.
